# Functional Characterization and Screening of Promiscuous Kinases and Isopentenyl Phosphate Kinases for the Synthesis of DMAPP via a One-Pot Enzymatic Cascade

**DOI:** 10.3390/ijms232112904

**Published:** 2022-10-26

**Authors:** Cong Qiu, Yang Liu, Yangbao Wu, Linguo Zhao, Jianjun Pei

**Affiliations:** 1 Jiangsu Co-Innovation Center of Efficient Processing and Utilization of Forest Resources, College of Chemical Engineering, Nanjing Forestry University, Nanjing 210037, China; 2 Jiangsu Key Lab of Biomass-Based Green Fuels and Chemicals, Nanjing 210037, China

**Keywords:** dimethylallyl diphosphate, promiscuous kinase, isopentenyl phosphate kinase, one-pot enzymatic cascade

## Abstract

Dimethylallyl diphosphate (DMAPP) is a key intermediate metabolite in the synthesis of isoprenoids and is also the prenyl donor for biosynthesizing prenylated flavonoids. However, it is difficult to prepare DMAPP via chemical and enzymatic methods. In this study, three promiscuous kinases from *Shigella flexneri* (SfPK), *Escherichia coli* (EcPK), and *Saccharomyces cerevisiae* (ScPK) and three isopentenyl phosphate kinases from *Methanolobus tindarius* (MtIPK), *Methanothermobacter thermautotrophicus* str. Delta H (MthIPK), and *Arabidopsis thaliana* (AtIPK) were cloned and expressed in *Escherichia coli*. The enzymatic properties of recombinant enzymes were determined. The *Kcat/Km* value of SfPK for DMA was 6875 s^−1^ M^−1^, which was significantly higher than those of EcPK and ScPK. The *Kcat/Km* value of MtIPK for DMAP was 402.9 s^−1^ M^−1^, which was ~400% of that of MthIPK. SfPK was stable at pH 7.0–9.5 and had a 1 h half-life at 65 °C. MtIPK was stable at pH 6.0–8.5 and had a 1 h half-life at 50 °C. The stability of SfPK and MtIPK was better than that of the other enzymes. Thus, SfPK and MtIPK were chosen to develop a one-pot enzymatic cascade for producing DMAPP from DMA because of their catalytic efficiency and stability. The optimal ratio between SfPK and MtIPK was 1:8. The optimal pH and temperature for the one-pot enzymatic cascade were 7.0 and 35 °C, respectively. The optimal concentrations of ATP and DMA were 10 and 80 mM, respectively. Finally, maximum DMAPP production reached 1.23 mM at 1 h under optimal conditions. Therefore, the enzymatic method described herein for the biosynthesis of DMAPP from DMA can be widely used for the synthesis of isoprenoids and prenylated flavonoids.

## 1. Introduction

Dimethylallyl diphosphate (DMAPP)—a kind of 5-carbon diphosphate—is a key intermediate metabolite in the synthesis of isoprenoids. Isoprenoids are a large and important class of natural products found in nature. To date, over 55,000 isoprenoids have been found in nature. Isoprenoids play important physiological, ecological, and pharmacological roles and have been used to develop pharmaceuticals, nutritional supplements, and fuels [1,2,3,4]. DMAPP is also the prenyl donor for biosynthesizing prenylated compounds through the action of prenyltransferase [5,6,7]. The prenylated compounds can increase their affinity to cell membranes and improve their interaction with the target protein by increasing the lipophilicity of natural compounds, endowing the natural compounds with greater bioactivity and bioavailability [8,9,10,11]. For example, icaritin—a kind of prenylated flavonoid extracted from *Epimedium* spp.—has been explored to treat advanced hepatocellular carcinoma. Therefore, the efficient preparation of DMAPP has become important [12,13,14].

In nature, two different metabolic pathways—the mevalonate (MVA) and methylerythritol phosphate (MEP) pathways—exist to biosynthesize DMAPP and its isomer isopentenyl pyrophosphate (IPP) [15,16,17,18,19]. The MVA metabolic pathway begins with the reactions that condense acetyl-CoA molecules to form 3-hydroxy-3-methylglutaryl-CoA (HMG-CoA) via the action of bifunctional acetoacetyl-CoA thiolase/3-hydroxy-3-methylglutar-yl-CoA (HMG-CoA) reductase (MvaE) and HMG-CoA synthase (MvaS). HMG-CoA is then converted to DMAPP through five consecutive reactions catalyzed by MvaE, mevalonate kinase, phosphomevalonate kinase, mevalonate pyrophosphate decarboxylase, and isopentenyl pyrophosphate isomerase [16]. In the MEP metabolic pathway, DMAPP is synthesized from glyceraldehyde 3-phosphate (GAP) and pyruvate through seven consecutive reactions catalyzed by 1-deoxy-D-xylulose5-phosphate synthase, DXP reductase, 4-hydroxy-3-methylbut-2-enyl-di-phosphate synthase, 4-hydroxy-3-methylbut-2-enyl-di-phosphate reductase, and isopentenyl pyrophosphate isomerase [16]. Recombinant strains have been developed to improve the supply of DMAPP and IPP by optimizing the MEP and MVA metabolic pathways in vivo [16,18,19]. Although DMAPP can also be synthesized through these consecutive enzymatic reactions, the seven enzymatic steps and cofactor regeneration make it challenging to develop an enzymatic cascade to produce DMAPP via the MVA and MEP pathways in vitro [16].

In recent years, an alternative metabolic pathway—termed the isopentenol utilization pathway (IUP)—has been developed to increase the supply of DMAPP or IPP in recombinant strains [20,21,22,23]. The IUP consists of two kinases—promiscuous kinase and isopentenyl phosphate kinase—which sequentially phosphorylate prenol into DMAPP via dimethylallyl phosphate (DMAP) intermediates. Thus, the IUP makes it possible to produce DMAPP though enzymatic methods [24,25,26,27,28,29]. Promiscuous kinases can be coupled with isopentenyl phosphate kinases to develop a simple and efficient enzymatic method that can use prenol as an inexpensive substrate for the synthesis of DMAPP in vitro, with ATP as the cofactor.

Compared to transformation via recombinant strains in vivo, multi-enzyme catalytic systems in vitro have the following advantages: reducing the reaction time, improving the tolerance to toxic compounds, preventing the degradation of substrates and products, and facilitating process control [30,31,32]. Some multi-enzyme catalytic systems have been successfully developed to biosynthesize natural compounds [33,34]. In this study, we report the cloning, expression, and characterization of three promiscuous kinases and three isopentenyl phosphate kinases from plants and microbes. A one-pot enzymatic cascade was developed for the production of DMAPP from DMA by using heterologously expressed *S. flexneri* promiscuous kinase (SfPK) and *M. tindarius* isopentenyl phosphate kinase (MtIPK) (Figure S1). Moreover, the optimal conditions for the enzymatic cascade were determined. 

## 2. Results and Discussion

### 2.1. Cloning and Expression of Promiscuous Kinases

**Figure 1:**
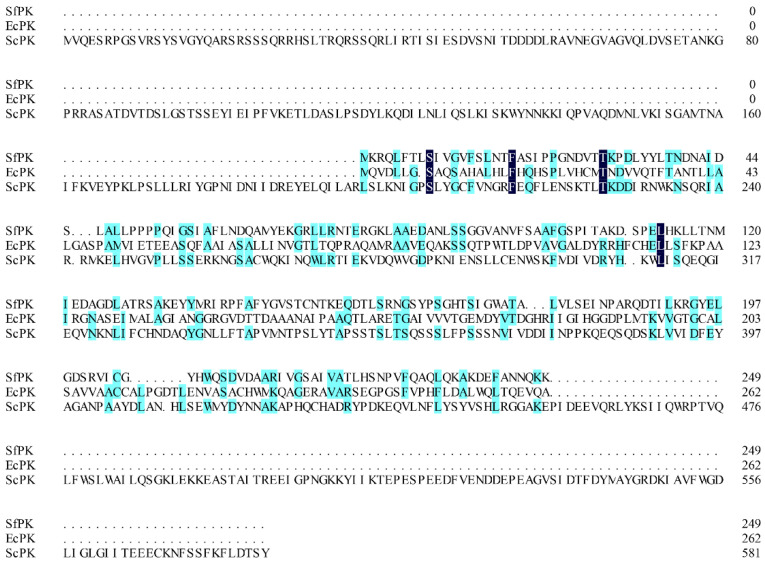
Alignment of promiscuous kinases. SfPK: *Shigella flexneri* promiscuous kinase, EcPK: *Escherichia coli* promiscuous kinase, ScPK: *Saccharomyces cerevisiae* promiscuous kinase.

Generally, screening and obtaining appropriate enzymes with high specific activity is a key factor in developing an efficient enzymatic reaction to biosynthesize natural compounds in vitro. Some promiscuous kinase genes from *S. flexneri* (*SfPK*), *E. coli* (*EcPK*), and *Saccharomyces cerevisiae* (*ScPK*) have been introduced into recombinant strains to reconstruct the IUP in order to improve the supply of DMAPP or IPP in vivo [20,21,22,23]. However, only a few promiscuous kinase genes have been expressed and characterized in heterologous hosts [24,35]. In this study, three promiscuous kinases (SfPK, EcPK, and ScPK) were chosen to characterize the biochemical properties. *SfPK*, *EcPK*, and *ScPK* consist of 750 bp, 789 bp, and 1749 bp fragments encoding 249, 262, and 582 amino acids, respectively. The alignment of these three promiscuous kinases showed that SfPK had 13.6% and 5.5% amino acid sequence homology with EcPK and ScPK, respectively (Figure 1). Thus, these results suggest that the enzymes could have different biochemical properties.

**Figure 2:**
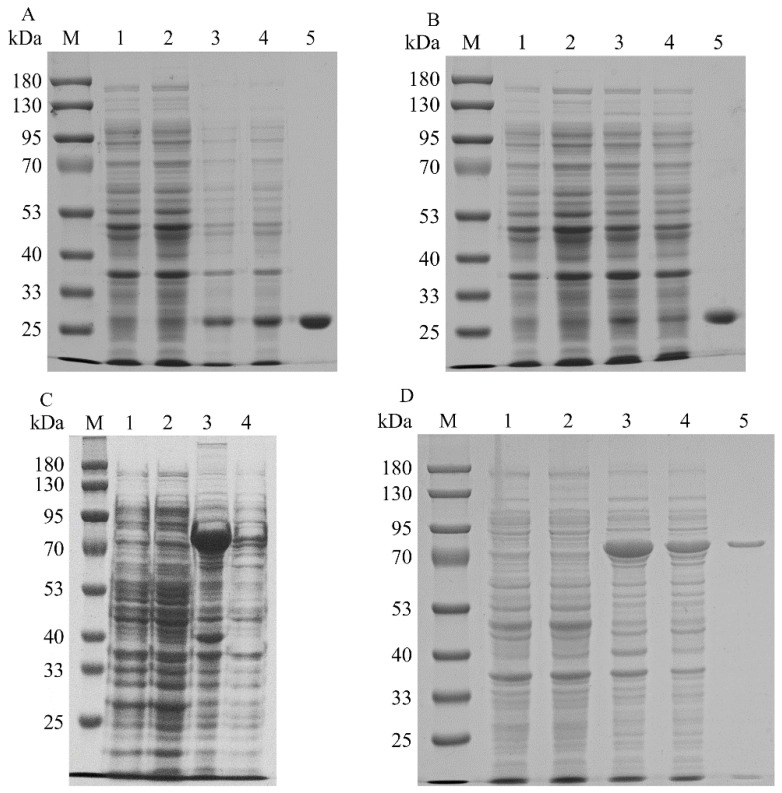
SDS–PAGE analysis of recombinant promiscuous kinases in *E. coli*: (**A**) SDS–PAGE analysis of SfPK (lane M: protein marker; lane 1: the total protein of BL21(DE3); lane 2: the soluble protein in BL21(DE3); lane 3: the total protein of BL2-SfPK; lane 4: the soluble protein in BL21-SfPK; lane 5: purified SfPK). (**B**) SDS–PAGE analysis of EcPK (lane M: protein marker; lane 1: the total protein of BL21(DE3); lane 2: the soluble protein in BL21(DE3); lane 3: the total protein of BL2-EcPK; lane 4: the soluble protein in BL21-EcPK; lane 5: purified EcPK). (**C**) SDS–PAGE analysis of ScPK without optimizing induction conditions (lane M: protein marker; lane 1: the total protein of BL21(DE3); lane 2: the soluble protein in BL21(DE3); lane 3: the total protein of BL2-ScPK; lane 4: the soluble protein in BL21-ScPK). (**D**) SDS–PAGE analysis of ScPK (lane M: protein marker; lane 1: the total protein of BL21(DE3); lane 2: the soluble protein in BL21(DE3); lane 3: the total protein of BL2-ScPK; lane 4: the soluble protein in BL21-ScPK; lane 5: purified ScPK).

To increase the expression levels of the promiscuous kinases, the codons of SfPK, EcPK, and ScPK were optimized to incorporate *E. coli* codons. SDS–PAGE analysis of the recombinant promiscuous kinases is shown in Figure 2. The recombinant ScPK was overexpressed by adding 0.1 mM IPTG at 30 °C for ~12 h. However, the overexpression of ScPK resulted in the production of a large number of inclusion bodies (Figure 2C). To reduce the inclusion body formation of ScPK, different induction strategies were adopted to express ScPK. Most of the enzyme was soluble in the cell-free extracts under induction at 30 °C with 0.1 mM IPTG in SOC medium for 4 h (Figure 2D) [20]. The recombinant promiscuous kinases were purified to electrophoretic purity by using Ni-NTA affinity. The molecular masses of SfPK, EcPK, and ScPK were estimated to be 27.4, 28.8, and 64 kDa, respectively (Figure 2A,B,D, respectively). SfPK was purified 5.9-fold with the specific activity of 0.19 µmol/min/mg, EcPK was purified 20-fold with the specific activity of 0.1 µmol/min/mg, and ScPK was purified 7.2-fold with the specific activity of 0.47 µmol/min/mg (Table 1).

### 2.2. Characterization of Recombinant Promiscuous Kinases

**Figure 3:**
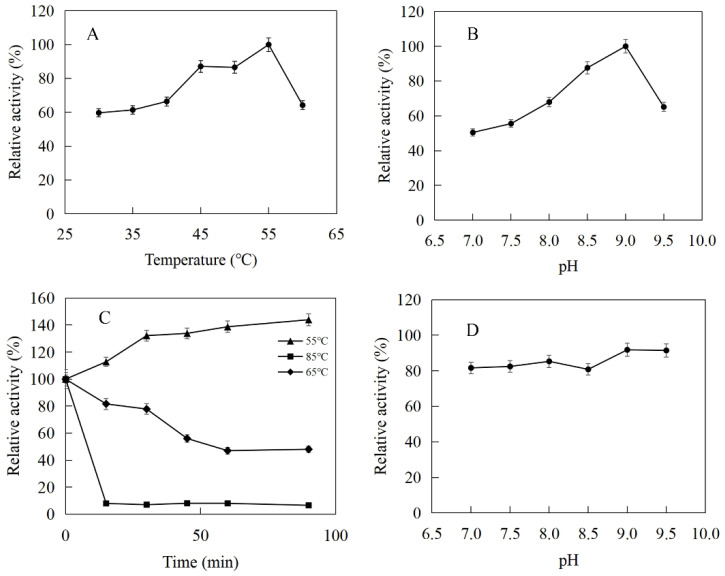
Effects of pH and temperature on the activity and stability of SfPK: (**A**) Effect of temperature on the activity of SfPK. (**B**) Effect of pH on the activity of SfPK. (**C**) Thermostability of SfPK. (**D**) pH stability of SfPK.

**Figure 4:**
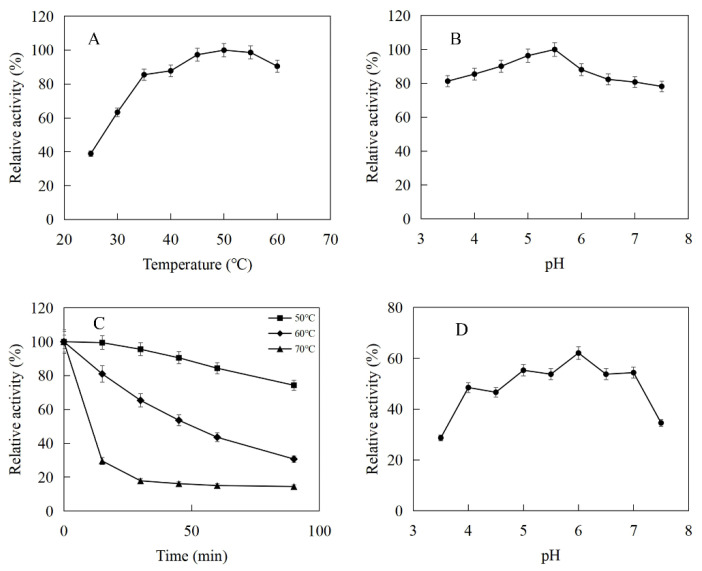
Effects of pH and temperature on the activity and stability of EcPK: (**A**) Effect of temperature on the activity of EcPK. (**B**) Effect of pH on the activity of EcPK. (**C**) Thermostability of EcPK. (**D**) pH stability of EcPK.

**Figure 5:**
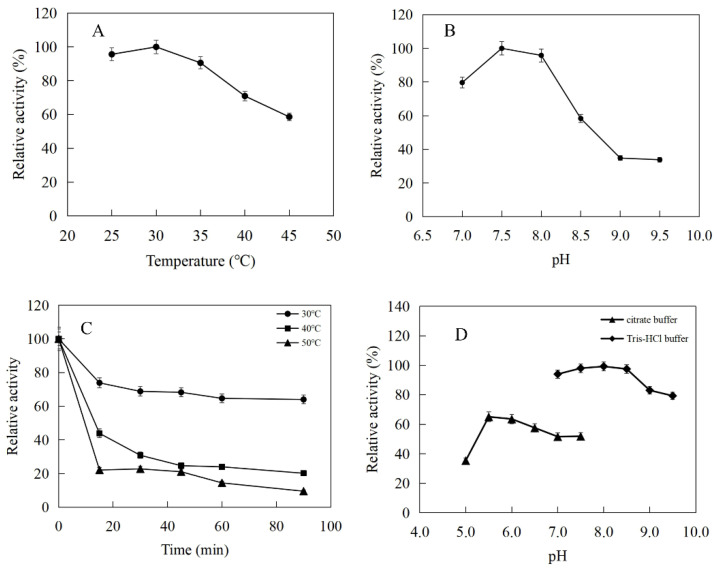
Effects of pH and temperature on the activity and stability of ScPK: (**A**) Effect of temperature on the activity of ScPK. (**B**) Effect of pH on the activity of ScPK. (**C**) Thermostability of ScPK. (**D**) pH stability of ScPK.

The enzymatic properties were investigated using the purified recombinant SfPK, EcPK, and ScPK. The optimal temperature of SfPK was at 55 °C, while the activity of SfPK in the temperature range of 30–55 °C exceeded 60% of the maximum activity (Figure 3A). The analysis of thermostability showed that the activity of SfPK increased by 140% after incubation at 55 °C for 90 min, and the residual activity of SfPK was 50% after incubation at 65 °C for 1 h (Figure 3C). The optimal pH of SfPK was determined to be 9.0, while the activity of SfPK in the pH range of 7.0–9.5 exceeded 50% of the maximum activity (Figure 3B). The enzyme was stable for ~12 h at pH 7.0–9.5 and 4 °C in the absence of the substrate (Figure 3D). The optimal temperature of EcPK was 50 °C, while the activity of EcPK in the temperature range of 35–60 °C exceeded 80% of the maximum activity (Figure 4A). The analysis of thermostability showed that the residual activity of EcPK was ~40% after incubation at 60 °C for 1 h (Figure 4C). The optimal pH of EcPK was determined to be 5.5, while the activity of EcPK in the pH range of 3.5–7.5 exceeded 70% of the maximum activity (Figure 4B). Recombinant EcPK was unstable for ~12 h at pH 3.5–7.5 and 4 °C in the absence of the substrate (Figure 4D). The optimal temperature of ScPK was 30 °C, while the activity of ScPK in the temperature range of 25–40 °C exceeded 60% of the maximum activity (Figure 5A). The analysis of thermostability showed that the residual activity of ScPK was ~20% after incubation at 40 °C for 1 h (Figure 5C). The optimal pH of ScPK was determined to be 7.5 (Figure 5B). Recombinant ScPK was more stable in Tris-HCl buffer than in citrate buffer (Figure 5D). These results show that the temperature and pH stability of SfPK are better than those of EcPK and ScPK.

The effects of metal ions and reagents on the activities of SfPK, EcPK, and ScPK are listed in Table 2. The activity of SfPK was significantly increased by Ba^2+^, Mg^2+^ and Al^3+^, while it was inhibited by Fe^3+^, Ni^2+^ and Zn^2+^. No metal irons increased the activity of EcPK, which was significantly inhibited by Fe^3+^, Ba^2+^, Fe^2+^ and EDTA. The activity of ScPK was slightly increased by Mg^2+^ and significantly inhibited by Fe^3+^, Co^2+^ and EDTA. In general, phosphokinases have the dimeric structure with two binding domains and can bind the nucleophilic phosphate group, Mg^2+^ and ATP. Although, SfPK had no Mg^2+^ binding domain by comparing the amino acid sequences, the activity of SfPK was significantly improved by adding Mg^2+^.

The dependence of the rate of the enzymatic reaction on the substrate concentration followed Michaelis–Menten kinetics. The *Km* and *Vmax* values of SfPK for DMA were 0.016 mM and 0.24 µmol/min/mg, respectively, under optimal conditions. The *Km* and *Vmax* values of EcPK for DMA were 0.93 mM and 0.13 µmol/min/mg, respectively, under optimal conditions. The *Km* and *Vmax* values of ScPK for DMA were 1.25 mM and 0.92 µmol/min/mg, respectively, under optimal conditions (Figure S2a–c). The *Kcat/Km* value of SfPK for DMA was 6875 s^−1^ M^−1^, which was significantly higher than those of EcPK (67.7 s^−1^ M^−1^) and ScPK (784 s^−1^ M^−1^) (Table S1). 

### 2.3. Cloning and Expression of Isopentenyl Phosphate Kinase

Isopentenyl phosphate kinases belong to the superfamily of amino acid kinases and can phosphorylate DMAP to produce DMAPP with ATP as the cofactor. Isopentenyl phosphate kinases all contained a superimposable histidine at position 60 (His60), which played a key role in stabilizing the terminal phosphate group of the product and substrate. Isopentenyl phosphate kinases catalyzed the phosphorylation reaction with a single displacement mechanism and no phosphoryl-enzyme intermediate was produced to interfere with the reaction [26,29].

**Figure 6:**
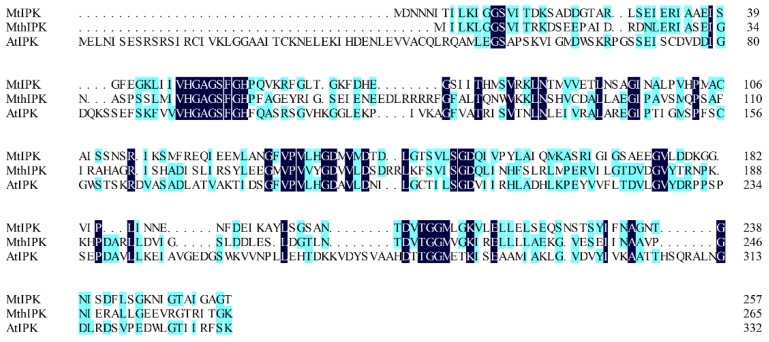
Alignment of isopentenyl phosphate kinases. MtIPK: *Methanolobus tindarius* isopentenyl phosphate kinase, MthIPK: *Methanothermobacter thermautotrophicus* str. Delta H isopentenyl phosphate kinase, AtIPK: *Arabidopsis thaliana* isopentenyl phosphate kinase.

Isopentenyl phosphate kinases from *M. tindarius* (MtIPK), *Methanothermobacter thermautotrophicus* str. Delta H (MthIPK), and *A. thaliana* (AtIPK) have been used to reconstruct the IUP in the recombinant strains for improvement of the supply of DMAPP [4,29,36,37,38,39]. Thus, in this study, MtIPK, MthIPK, and AtIPK were chosen to characterize the biochemical properties. *MtIPK*, *MthIPK*, and *AtIPK* consist of 777 bp, 801 bp, and 999 bp fragments encoding 258, 266, and 332 amino acids, respectively. The alignment of these three isopentenyl phosphate kinases indicated that they belong to the amino acid kinase family. Sequence alignments of the three isopentenyl phosphate kinases showed that MtIPK had 15.7% and 12.8% amino acid sequence homology with MthIPK and AtIPK, respectively (Figure 6).

**Figure 7:**
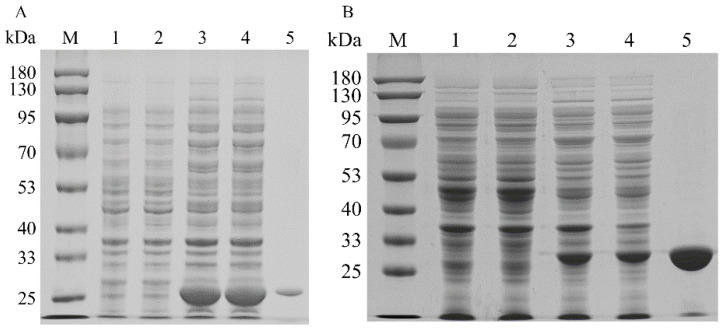
SDS–PAGE analysis of recombinant isopentenyl phosphate kinases in *E. coli*: (**A**) SDS–PAGE analysis of MtIPK (lane M: protein marker; lane 1: the total protein of BL21(DE3); lane 2: the soluble protein in BL21(DE3); lane 3: the total protein of BL2-MtIPK; lane 4: the soluble protein in BL21-MtIPK; lane 5: purified MtIPK). (**B**) SDS–PAGE analysis of MthIPK (lane M: protein marker; lane 1: the total protein of BL21(DE3); lane 2: the soluble protein in BL21(DE3); lane 3: the total protein of BL2-MthIPK; lane 4: the soluble protein in BL21-MthIPK; lane 5: purified MthIPK).

To increase the expression levels of the isopentenyl phosphate kinases, the codons of MtIPK, MthIPK, and AtIPK were optimized to incorporate *E. coli* codons. The SDS–PAGE analysis of the recombinant isopentenyl phosphate kinases is shown in Figure 7. The recombinant isopentenyl phosphate kinases were induced by adding 0.1 mM IPTG at 20 °C. However, the overexpression of AtIPK resulted in the production of large numbers of inclusion bodies. Molecular chaperones were introduced to reduce the inclusion body formation of AtIPK using the plasmids pGro7, pG-KJE8, pTf16, and pKJE7 [40]. However, the soluble expression of AtIPK in these recombinant strains did not improve. Finally, no purified AtIPK was obtained using Ni-NTA affinity. The recombinant MtIPK and MthIPK were purified to electrophoretic purity using Ni-NTA affinity (Figure 7A,B). The molecular masses of MtIPK and MthIPK were estimated to be 28.3 and 29.2, respectively (Figure 7A,B). MtIPK was purified 8-fold with the specific activity of 0.045 µmol/min/mg, while MthIPK was purified 16-fold with the specific activity of 0.016 µmol/min/mg (Table 1).

### 2.4. Characterization of Recombinant Isopentenyl Phosphate Kinase

**Figure 8:**
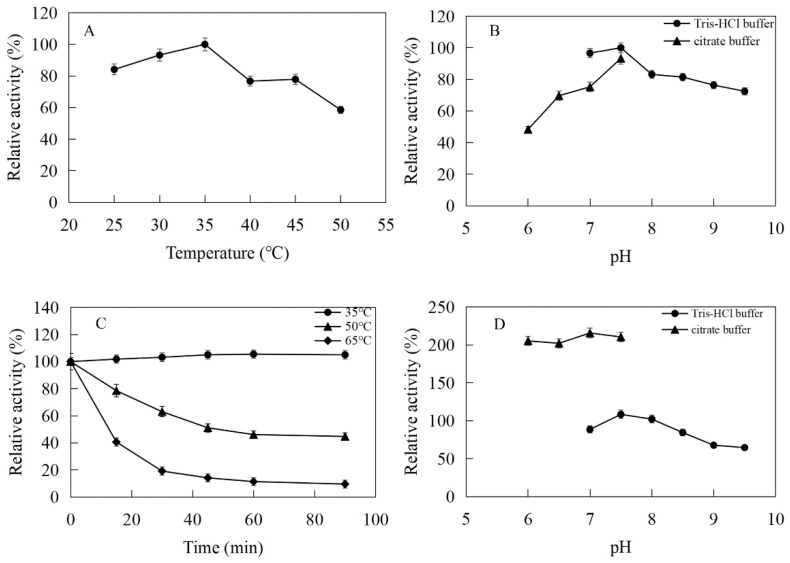
Effects of pH and temperature on the activity and stability of MtIPK: (**A**) Effect of temperature on the activity of MtIPK. (**B**) Effect of pH on the activity of MtIPK. (**C**) Thermostability of MtIPK. (**D**) pH stability of MtIPK.

**Figure 9:**
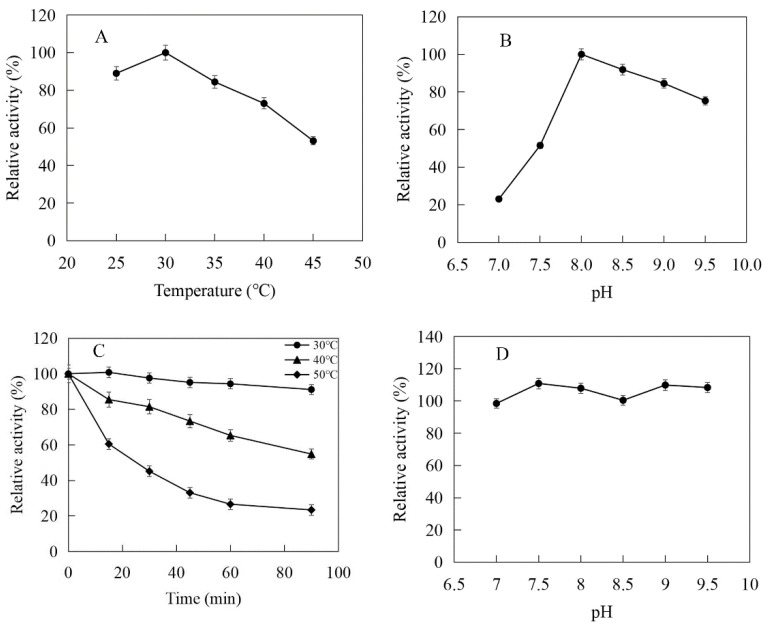
Effects of pH and temperature on the activity and stability of MthIPK: (**A**) Effect of temperature on the activity of MthIPK. (**B**) Effect of pH on the activity of MthIPK. (**C**) Thermostability of MthIPK. (**D**) pH stability of MthIPK.

The enzymatic properties were investigated using the purified recombinant MtIPK and MthIPK. The optimal temperature of MtIPK was 35 °C, while the activity of MtIPK in the temperature range of 25–45 °C exceeded 70% of the maximum activity (Figure 8A). The analysis of thermostability showed that the residual activity of MtIPK was 50% after incubation at 50 °C for 1 h (Figure 8C). The optimal pH of MtIPK was determined to be 7.5, while the activity of MtIPK in the pH range of 7.0–9.5 exceeded 60% of the maximum activity. The activity of MtIPK in the Tris-HCl buffer was higher than that in citric acid buffer under the same pH value (Figure 8B). The enzyme was stable for 12 h at pH 6.0–8.5 and 4 °C in the absence of the substrate (Figure 8D). The activity of MtIPK was significantly increased when the enzyme was incubated for 12 h in citric acid buffer (pH 6.0–7.0) at 4 °C (Figure 8D). Citric acid buffer was commonly used to assay the activity of enzyme, but no literature reported that it can improve the activity of isopentenyl phosphate kinase. This is the first observation of citrate improving the activity of isopentenyl phosphate kinase, and the reasons are yet to be determined. The optimal temperature of MthIPK was 30 °C (Figure 9A). The analysis of thermostability showed that the residual activity of MthIPK was 60% after incubation at 40 °C for 1 h (Figure 9C). The optimal pH of MthIPK was determined to be 7.5 (Figure 9B). The enzyme was stable for 12 h at pH 7.0–9.5 and 4 °C in the absence of the substrate (Figure 9D).

The effects of metal ions and reagents on the activities of MtIPK and MthIPK are listed in Table 3. The activity of MtIPK was significantly inhibited by Sr^2+^, Co^2+^, K^+^ and EDTA; the effects of Ca^2+^, Mn^2+^ and Mg^2+^ on the activity of MtIPK were not as significant. The activity of MthIPK was significantly inhibited by EDTA; the effects of Fe^3+^, K^+^, Ba^2+^, Sr^2+^, Ca^2+^, Cu^2+^, Ni^2+^, Co^2+^, Zn^2+^, Mn^2+^, Na^+^, Fe^2+^, Li^+^, Mg^2+^, Al^3+^ and NH^4+^ on the activity of MthIPK were not as significant. 

The dependence of the rate of the enzymatic reaction on the substrate concentration followed Michaelis–Menten kinetics. The *Km* and *Vmax* values of MtIPK for DMAP were 0.065 mM and 0.059 µmol/min/mg, respectively, under optimal conditions. The *Km* and *Vmax* values of MthIPK for DMAP were 0.116 mM and 0.024 µmol/min/mg, respectively, under optimal conditions (Figure S2d,e). The *Kcat/Km* value of MtIPK for DMAP was 402.9 s^−1^ M^−1^, which was ~400% of that of MthIPK (100.9 s^−1^ M^−1^) (Table S1). These results show that the catalytic efficiency of the isopentenyl phosphate kinases is significantly lower than that of SfPK, which is important for developing the IUP in vivo and in vitro to improve the catalytic efficiency of isopentenyl phosphate kinases.

### 2.5. Developing a One-Pot Enzymatic Cascade for the Synthesis of DMAPP

**Figure 10:**
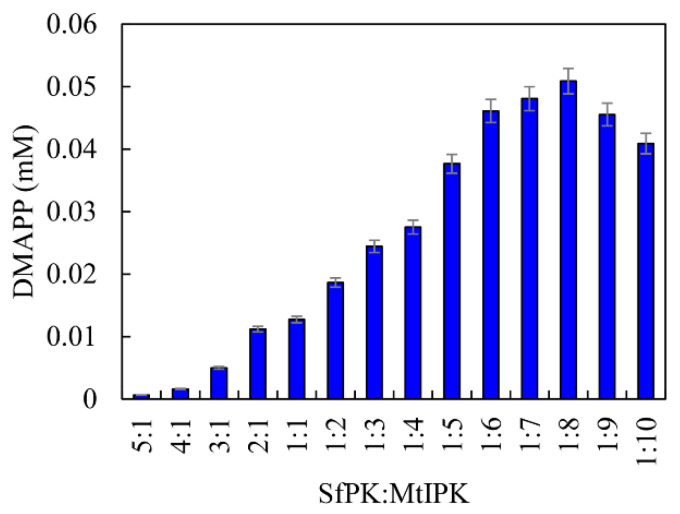
Effect of enzyme concentration on the production of DMAPP via the one-pot enzymatic cascade.

From the above results, it can be seen that SfPK and MtIPK represent potent candidates for the synthesis of DMAPP in terms of their catalytic efficiency and stability. Because the *Kcat/Km* value of SfPK was 1576% of that of MtIPK, it is important to regulate the catalytic efficiency of enzymes in vitro when developing the one-pot enzymatic cascade. The catalytic efficiencies of enzymes can be easily matched by changing the ratios of the enzymes [33]. The effects of the ratio between SfPK and MtIPK on DMAPP production were determined, as shown in Figure 10. DMAPP production increased by 299% when the ratio between MtIPK and SfPK increased from 1:1 to 8:1, whereas DMAPP production decreased when the ratio between SfPK and MtIPK increased from 1:1 to 5:1. The results indicated that the one-pot synthesis of DMAPP catalyzed by MtIPK was the rate-limiting step in the present system, and the optimal ratio between SfPK and MtIPK was 1:8.

Because it is difficult to directly detect DMAPP production in the one-pot enzymatic cascade, DMAPP production was determined by detecting the concentration of 7-dimethylallyl tryptophan, which was transformed from tryptophan by prenyltransferase (7-DMATS). HPLC and LC–MS of 7-dimethylallyl tryptophan and tryptophan have been provided in Figure S3. The m/z value of the molecular ion [M-H]- of the one-pot enzymatic product was 271.1460, which was equal to the m/z value of 7-dimethylallyl tryptophan. This results showed that DMAPP was produced from DMA by the one-pot enzymatic cascade.

### 2.6. Optimizing the Conditions of the One-Pot Enzymatic Cascade

**Figure 11:**
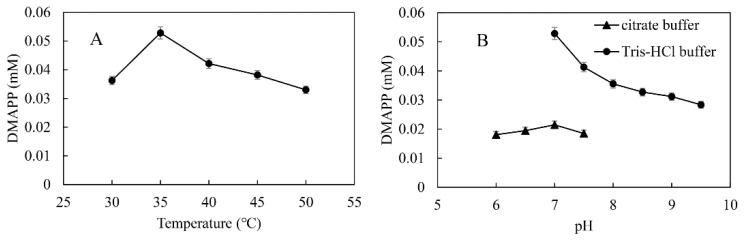
Optimization of conditions for the production of DMAPP via the one-pot enzymatic cascade: (**A**) Effect of temperature on DMAPP production. (**B**) Effect of pH on DMAPP production.

Temperature and pH are important factors in the synthesis of DMAPP because they affect the activity and stability of SfPK and MtIPK. The optimal temperature and pH for the one-pot enzymatic cascade were 35 °C and 7.0, respectively—the same as those of MtIPK, and well below those of SfPK (Figure 11). These results indicate that it is important to improve the activity of MtIPK for the synthesis of DMAPP. The production of DMAPP in Tris-HCl buffer was significantly higher than that in citric acid buffer at the same pH. The result suggested that citrate could have a negative effect on the activity of SfPK. Thus, Tris-HCl buffer (pH 7.0) and a temperature of 35 °C were used for the one-pot enzymatic cascade.

**Figure 12:**
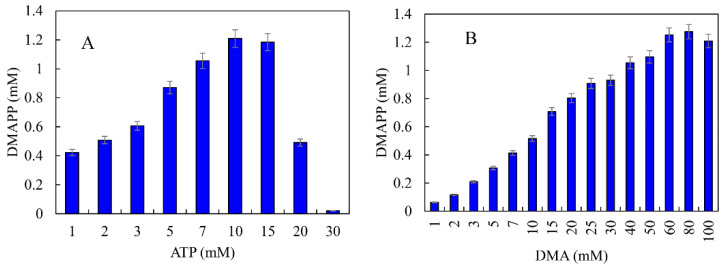
Effects of (**A**) ATP and (**B**) DMA on the production of DMAPP via the one-pot enzymatic cascade.

**Figure 13:**
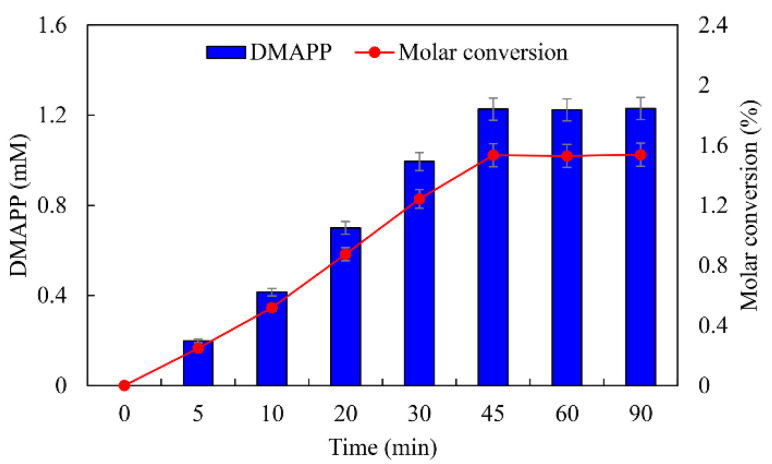
Time course for the production of DMAPP under optimal conditions.

ATP was an important cofactor for the synthesis of DMAPP [26,36]. The production of DMAPP increased linearly when the concentration of ATP increased from 1 to 10 mM, while it decreased when the concentration of ATP exceeded 10 mM. DMAPP production was only 0.49 mM when the concentration of ATP reached 20 mM (Figure 12A). These results indicate that high concentrations of ATP could inhibit the one-pot enzymatic cascade. The effects of DMA on the synthesis of DMAPP were determined. The production of DMAPP increased linearly when the concentration of DMA increased from 1 to 60 mM. The highest production of DMAPP reached 1.21 mM with the addition of 80 mM DMA (Figure 12B). Similar results have been reported with the recombinant strains, and the highest yields were observed using 20~25 mM prenol in vivo [20,39]. The half-saturation constant of DMA for the one-pot enzymatic cascade was ~12 mM—significantly higher than the *Km* values of SfPK and MtIPK. These results indicate that the supply of DMA is the key factor for the production of DMAPP via the IUP in vivo or in vitro. Under the optimal conditions, the time course for the production of DMAPP was as shown in Figure 13. The specific productivity for DMAPP was 33.1 μM/min during the reaction time from 0 to 30 min. Then, the specific productivity gradually decreased as the reaction proceeded, and the specific productivity for DMAPP was only 15.5 μM/min over a reaction time of 30–45 min. After 1 h, the reaction reached equilibrium, and the maximum DMAPP production reached 1.23 mM. Although promiscuous kinases and isopentenyl phosphate kinases have been used to increase the supply of DMAPP or IPP in vivo [20,21,22,23], this is the first time to develop a one-pot enzymatic cascade to produce DMAPP in vitro.

## 3. Materials and Methods

### 3.1. Strains, Plasmids, Media and Chemicals

All of the plasmids and strains used in this research are listed in Table 4. The recombinant strains were grown at 37 °C in Luria–Bertani (LB) medium supplemented with antibiotics when required. Prenol and 3-methyl-2-buten-1-ol (DMA) were purchased from Shanghai Aladdin Bio-Chem Technology Co., Ltd. (Shanghai, China). ADP·Na_2_, PEP-K, and β-NADH were purchased from Sangon Biotech Co., Ltd. (Shanghai, China). Pyruvate kinase and lactic dehydrogenase were purchased from Sigma-Aldrich Chemical Co. (St. Louis, MO, USA). L-tryptophan and other conventional reagents were purchased from Sinopharm Chemical Reagent Co., Ltd. (Shanghai, China). The SOC medium contained 20 g/L tryptone, 5 g/L yeast extract, 10 mM NaCl, 2.5 mM KCl, 10 mM MgCl_2_, 20 mM MgSO_4_, and 20 mM glucose.

### 3.2. Plasmid Construction

*SfPK* (GenBank No. D82966.1), *EcPK* (GenBank No. NP_416607.1), *MtIPK* (GenBank No. WP_023846034.1), and *MthIPK* (GenBank No. NC_000916.1) were synthesized by incorporating the *E. coli* codons. The Nco I site was added to the 5′ end of the genes. The BamH I site and six histidine residues were added to the 3′ end of the genes. SfPK, EcPK, MtIPK, and MthIPK were digested with Nco I and BamH I, and then subcloned into the expression vector pCDFDuet-1 at the Nco I and BamH I sites to create pCDFDuet-SfPK, pCDFDuet-EcPK, pCDFDuet-MtIPK, and pCDFDuet-MthIPK, respectively. *ScPK* (GenBank No. J04454.1) and *AtIPK* (GenBank No. NM_102426.6) were synthesized by incorporating the *E. coli* codons. The Nco I site was added to the 5′ end of the genes, and the Xho I site was added to the 3′ end of the genes. *ScPK* and *AtIPK* were digested with Nco I and Xho I, and then subcloned into the expression vector pET-28a at the Nco I and Xho I sites to create pET-28a-ScPK and pET-28a-AtIPK, respectively. *7-DMATS* (GenBank No. EF539173.1) was synthesized by incorporating the *E. coli* codons. The Nde I site was added to the 5′ end of the gene. The Xho I site and six histidine residues were added to the 3′ end of the gene. 7-DMATS was digested with Nde I and Xho I, and then subcloned into the expression vector pETDuet-1 at the Nde I and Xho I sites to create pETDuet-7-DMATS.

### 3.3. Heterologous Expression and Purification of Recombinant Enzymes

The plasmids pCDFDuet-SfPK, pCDFDuet-EcPK, pET-28a-ScPK, pCDFDuet-MtIPK, pCDFDuet-MthIPK, pET-28a-AtIPK, and pETDuet-7-DMATS were transformed into *E. coli* BL21 (DE3) to obtain the recombinant strains BL21-SfPK, BL21-EcPK, BL21-ScPK, BL21-MtIPK, BL21-MthIPK, BL21-AtIPK, and BL21-7-DMATS, respectively. The plasmids pET-28a-AtIPK and pG-KJE8 were co-transformed into *E. coli* BL21 (DE3) to obtain the recombinant strain BL21-AtIPK-KJE8. The plasmids pET-28a-AtIPK and pGro7 were co-transformed into *E. coli* BL21 (DE3) to obtain the recombinant strain BL21-AtIPK-pGro7. The plasmids pET-28a-AtIPK and pKJE7 were co-transformed into *E. coli* BL21 (DE3) to obtain the recombinant strain BL21-AtIPK-pKJE7. The plasmids pET-28a-AtIPK and pTf16 were co-transformed into *E. coli* BL21 (DE3) to obtain the recombinant strain BL21-AtIPK-pTf16.

Seed culture (5 mL) was added to 500 mL of fresh LB medium with appropriate antibiotics. The recombinant strains were induced to express the recombinant SfPK, EcPK, MtIPK, MthIPK, and 7-DMATS by adding isopropyl-β-d-thiogalactopyranoside (IPTG) to a final concentration of 0.1 mM at an OD_600_ of approximately 0.8 before incubation at 30 °C for approximately 12 h. For the recombinant strain BL21-ScPK, the seed culture (5 mL) was added to 500 mL of fresh SOC medium with appropriate antibiotics. Cultures were incubated at 37 °C and then induced to express recombinant ScPK by adding IPTG to a final concentration of 0.1 mM until the OD_600_ reached 0.5. The induction was carried out at 25 °C for 4 h. For the recombinant strains BL21-AtIPK, BL21-AtIPK-KJE8, BL21-AtIPK-pGro7, BL21-AtIPK-pKJE7, and BL21-AtIPK-pTf16, the seed culture (2 mL) was added to 200 mL of fresh LB medium with appropriate antibiotics. Cultures were incubated at 37 °C until the OD_600_ reached 0.6–0.8, and then induced to express recombinant AtIPK by adding 0.1 mM IPTG, 0.5 g/L L-arabinose or 5 mg/L tetracycline. The induction was carried out at 20 °C for 12 h. The cells were harvested by centrifugation at 6000× *g* for 15 min and lysed by ultrasound. The supernatants were loaded onto the Ni-NTA affinity column (Sangon Biotech, Shanghai) and eluted with elution buffer (200 mM imidazole, 0.5 M NaCl, and 20 mM Tris-HCl (pH 7.8)). Proteins were examined by SDS–PAGE. Protein concentrations were determined using the Bradford method, with bovine serum albumin (BSA) as a standard.

### 3.4. Promiscuous Kinase Assay

The reaction mixture contained 2 mM DMA as the substrate, 2 mM ATP as a coenzyme factor, 50 mM citrate buffer or Tris-HCl buffer, 2 mM MgCl_2_, and purified recombinant protein (40 μg) in a total volume of 100 μL. The reaction mixture was incubated at a suitable temperature for 20 min and terminated by boiling for 3 min. The samples were used to analyze the ADP concentration in order to calculate the production of DMAP. The concentration of ADP was determined using the ADP Colorimetric/Fluorometric Assay Kit (Sigma-Aldrich, St. Louis, MO, USA). The control was prepared according to the reaction without the recombinant promiscuous kinases.

### 3.5. Isopentenyl Phosphate Kinase Assay

The reaction mixture contained 0.5 mM DMAP as the substrate, 2 mM ATP as a coenzyme factor, 50 mM citrate buffer or Tris-HCl buffer, 2 mM MgCl_2_, and purified recombinant protein (60 μg) in a total volume of 150 μL. The reaction mixture was incubated at a suitable temperature for 20 min and terminated by boiling for 3 min. The samples were used to analyze the ADP concentration in order to calculate the production of DMAPP. The control was prepared according to the reaction without the recombinant isopentenyl phosphate kinases. DMAP was prepared via the action of SfPK. The reaction mixture contained 2 mM DMA as the substrate, 4 mM ATP as a coenzyme factor, 50 mM Tris-HCl buffer (pH 9.0), 2 mM MgCl_2_, and purified recombinant protein (80 μg) in a total volume of 100 μL. The concentration of DMAP was determined by detecting the concentration of ADP in the reaction. The reaction mixture was incubated at 50 °C for 30 min and terminated by boiling for 3 min. The Michaelis–Menten equation was used to determine the apparent *Km* and *Kcat* values of the enzymes using the OriginPro software [41].

### 3.6. Determination of Enzyme Activities and Properties

The optimal pH for the activity was determined by incubation at an appropriate temperature for 20 min in citrate buffer (50 mM, pH 2.5–7.5) or Tris-HCl buffer (50 mM, pH 7.0–9.5). The optimal temperature was determined using a standard assay with a temperature ranging from 5 to 60 °C. The pH stability of these enzymes was determined by measuring the residual activity after incubating the enzymes at 4 °C for 12 h in citrate buffer from pH 2.5 to 7.5 and Tris-HCl buffer from pH 7.0 to 9.5. The temperature stability of these enzymes was measured by determining the residual activity after incubating the enzymes for different times at different temperatures in the absence of the substrate. The effects of metals and chemical agents on the activities of the enzymes were determined. Then, 1 mM metal ions (Mg^2+^, Li^+^, K^+^, Ba^2+^, Zn^2+^, Na^+^, NH_4_^+^, Ca^2+^, Mn^2+^, Ca^2+^, Mn^2+^, Sr^2+^, Ni^2+^, Cu^2+^, Fe^2+^, Fe^3+^, Al^3+^ and Co^2+^) or 10 mM ethylenediaminetetraacetic acid (EDTA) was incubated with the purified recombinant protein, and the residual activity of the recombinant enzymes was measured.

### 3.7. Optimizing the Conditions of the Biocatalytic Cascade to Produce DMAPP

The standard reaction mixture contained 2 mM DMA, 4 mM ATP, 2 mM MgCl_2_, 50 mM Tris-HCl buffer (pH 7.0), and two enzymes with a total mass of 40 μg in a total volume of 100 μL, and was incubated for 1 h at 35 °C. The mixture was terminated by boiling for 3 min. 7-DMATS (60 μg) and 5 mM L-tryptophan were then added to produce 7-dimethylallyl tryptophan with DMAPP as the prenyl donor. The production of DMAPP was determined by detecting the concentration of 7-dimethylallyl tryptophan. The reaction mixture was incubated at 45 °C for 1 h and terminated by the addition of 200 μL methanol (MeOH). The protein was removed by centrifugation at 12,000× *g* for 5 min. 7-Dimethylallyl tryptophan was analyzed using high-performance liquid chromatography (HPLC).

To determine the optimal temperature, a standard assay with temperatures ranging from 30 to 50 °C was carried out in Tris-HCl buffer (50 mM, pH 7.0). The optimal pH for the biocatalytic cascade was determined by incubation at 35 °C in citrate buffer (50 mM, pH 6.0–7.5) and Tris-HCl buffer (50 mM, pH 7.0–9.5). The optimal ratio between SfPK and MtIPK was determined via a standard assay by adding different ratios of SfPK and MtIPK. The effects of DMA (1, 2, 3, 5, 7, 10, 15, 20, 25, 30, 40, 50, 60, 80 or 100 mM) and ATP (1, 2, 3, 5, 7, 10, 15, 20, or 30 mM) on the biocatalytic cascade were determined.

### 3.8. HPLC and Liquid Chromatography–Mass Spectrometry (LC–MS) Analyses

The Agilent HPLC series 1260 was used for the analysis of L-tryptophan and 7-dimethylallyl tryptophan. A Develosil RPAQUEOUS reverse-phase column (250 mm × 4.6 mm, 3.5 μm; Nomura Chemical Co., Hinode-Cho, Japan) was used for analysis at a flow rate of 1 mL/min. Acetonitrile (solvent B) and distilled water (solvent A) were used as solvents. The elution program was as follows: 0–20 min, 15–60% A; 20–25 min, 15% A. The detection was performed by monitoring the absorbance at 277 nm. LC–MS was performed for L-tryptophan and 7-dimethylallyl tryptophan using an LTQ Orbitrap XL LC–MS in negative mode and an ion trap analyzer. The ion spray was operated at 25 Arb N_2_/min, 3.5 kV, and 300 °C.

### 3.9. Statistical Analysis

Data are expressed as mean ± SD of three independent experiments. Student’s *t*-test and one-way ANOVA were used for statistical analysis of the data.

## 4. Conclusions

In this study, we reported the cloning and expression of three flavone synthases (SfPK, EcPK and ScPK) and three isopentenyl phosphate kinases (MtIPK, MthIPK and AtIPK). The enzymatic properties of SfPK, EcPK, ScPK, MtIPK and MthIPK were determined. The specific activity and stability of SfPK and MtIPK were better than those of other enzymes. SfPK was coupled with MtIPK to develop a one-pot enzymatic cascade for biosynthesizing DMAPP from DMA in vitro. By optimizing the conversion conditions, the maximum DMAPP production reached 1.23 mM at 1 h. This enzymatic cascade provides a widely available approach for the biosynthesis of DMAPP from DMA.

## Figures and Tables

**Table 1:** Purification of SfPK, EcPK, ScPK, MtIPK, and MthIPK from the recombinant strains.

Enzyme	Step	Total Protein (mg)	Total Activity (U)	Specific Activity (U/mg)	Yield (%)
SfPK	Cell extract	478	15.3	0.032	100
	Ni-NTA affinity	56	10.7	0.19	69.9
EcPK	Cell extract	452	2.3	0.005	100
	Ni-NTA affinity	13	1.3	0.1	56.6
ScPK	Cell extract	278	18.1	0.065	100
	Ni-NTA affinity	18	8.5	0.47	46.9
MtIPK	Cell extract	489	2.7	0.0056	100
	Ni-NTA affinity	4	0.18	0.045	6.6
MthIPK	Cell extract	463	0.5	0.001	100
	Ni-NTA affinity	21	0.34	0.016	68

**Table 2:** Effects of cations and EDTA on the activity of SfPK, EcPK, and ScPK.

Cation	Residual Activity (%)		
	SfPK	EcPK	ScPK
Control	100 ± 0.8	100 ± 0.4	100 ± 0.8
Fe^3+^	46.8 ± 2.1	5.8 ± 0.5	32.9 ± 1.1
K^+^	113.7 ± 1.2	79.1 ± 1.5	89.6 ± 0.8
Ba^2+^	138.9 ± 3.1	46.9 ± 0.9	56.2 ± 2.6
Sr^2+^	122.1 ± 1.6	66.6 ± 1.2	63.9 ± 1.9
Ca^2+^	99.4 ± 0.4	64.3 ± 0.6	96.4 ± 0.4
Cu^2+^	102.1 ± 0.8	68.4 ± 1.8	79.2 ± 0.8
Ni^2+^	32.5 ± 1.4	87.5 ± 1.1	64.6 ± 0.7
Co^2+^	105.7 ± 0.8	84.5 ± 0.8	33.2 ± 1.5
Zn^2+^	32.5 ± 1.0	44.6 ± 1.2	70.7 ± 1.8
Mn^2+^	90.0 ± 1.1	79.8 ± 0.5	81.2 ± 1.6
Na^+^	110.5 ± 0.5	81.5 ± 1.1	106.9 ± 0.6
Fe^2+^	80.5 ± 0.3	34.9 ± 0.9	52.6 ± 1.5
Li^+^	102.1 ± 0.9	81.5 ± 1.3	62.8 ± 0.9
Mg^2+^	182.9 ± 1.9	88.1 ± 1.2	113.8 ± 0.6
Al^3+^	152.5 ± 1.4	86.3 ± 1.4	77.2 ± 1.4
NH_4_^+^	118.5 ± 0.7	82.6 ± 0.8	73.5 ± 1.2
EDTA	96.3 ± 0.9	48.1 ± 0.9	46.1 ± 0.5

**Table 3:** Effects of cations and EDTA on the activities of MtIPK and MthIPK.

Cation	Residual Activity (%)	
	MtIPK	MthIPK
Control	100 ± 0.9	100 ± 1.1
Fe^3+^	82.9 ± 1.1	101.6 ± 0.3
K^+^	44.6 ± 0.5	104.2 ± 1.0
Ba^2+^	96.0 ± 0.8	109.8 ± 0.7
Sr^2+^	41.1 ± 0.2	87.0 ± 0.9
Ca^2+^	104.0 ± 1.3	118.1 ± 1.5
Cu^2+^	77.7 ± 0.8	105.7 ± 0.8
Ni^2+^	86.3 ± 1.2	109.8 ± 1.2
Co^2+^	46.9 ± 1.6	102.6 ± 0.6
Zn^2+^	84.0 ± 0.5	106.2 ± 0.5
Mn^2+^	95.4 ± 1.8	103.6 ± 0.4
Na^+^	77.1 ± 1.1	105.7 ± 1.4
Fe^2+^	58.9 ± 0.7	104.7 ± 0.8
Li^+^	61.1 ± 0.4	105.2 ± 0.6
Mg^2+^	101.7 ± 1.8	116.6 ± 1.3
Al^3+^	76.0 ± 0.6	108.8 ± 0.5
NH_4_^+^	66.3 ± 0.7	90.2 ± 0.7
EDTA	29.1 ± 1.6	46.1 ± 1.0

**Table 4:** Plasmids and strains used in this study.

Plasmid/Strain	Description	References
Plasmids		
pCDFDuet-SfPK	pCDFDuet-1 carrying *SfPK* from *Shigella flexneri*. T7 promoter, CloDF13 ori; streptomycin^r^	This study
pCDFDuet-EcPK	pCDFDuet-1 carrying *EcPK* from *Escherichia coli* str. K-12 substr. MG1655. T7 promoter, CloDF13 ori; streptomycin^r^	This study
pET-28a-ScPK	pET-28a (+) carrying *ScPK* from *Saccharomyces cerevisiae*. T7 promoter, pBR322 ori; kanamycin^r^	This study
pCDFDuet-MtIPK	pCDFDuet-1 carrying *MtIPK* from *Methanolobus tindarius*. T7 promoter, CloDF13 ori; streptomycin^r^	This study
pCDFDuet-MthIPK	pCDFDuet-1 carrying *MthIPK* from *Methanothermobacter thermautotrophicus* str. Delta H. T7 promoter, CloDF13 ori; streptomycin^r^	This study
pET-28a-AtIPK	pET-28a (+) carrying *AtIPK* from *Arabidopsis thaliana*. T7 promoter, pBR322 ori; kanamycin^r^	This study
pETDuet-7-DMATS	pETDuet-1 carrying *7-DMATS* from *Aspergillus fumigatus*. T7 promotor; ColE1 ori; ampicillin^r^	This study
pG-KJE8	Carrying chaperone protein DnaK gene (*dnaK*), chaperone protein DnaJ (*dnaJ*), chaperone protein GrpE gene (*grpE*), chaperone protein GroES gene (*groES*), and chaperone protein GroEL gene (*groEL*); *dnaK*, *dnaJ*, and *grpE* were promoted by the araB promotor; *grpES* and *groEL* were promoted by the tetR promoter	TaKaRa Bio ^a^
pGro7	Carrying the *groES* and *groEL* genes; *groES* and *groEL* were promoted by the araB promoter	TaKaRa Bio
pKJE7	Carrying the *dnaK*, *dnaJ*, and *grpE* genes; *dnaK*, *dnaJ*, and *grpE* were promoted by the araB promoter	TaKaRa Bio
pTf16	Carrying the trigger factor gene *tig*; *tig* was promoted by the araB promoter	TaKaRa Bio
Strains	*Escherichia coli* BL21 (DE3)	New England Biolabs ^b^
BL21-SfPK	BL21 (DE3) harboring pCDFDuet-SfPK	This study
BL21-EcPK	BL21 (DE3) harboring pCDFDuet-EcPK	This study
BL21-ScPK	BL21 (DE3) harboring pET-28a-ScPK	This study
BL21-MtIPK	BL21 (DE3) harboring pCDFDuet-MtIPK	This study
BL21-MthIPK	BL21 (DE3) harboring pCDFDuet-MthIPK	This study
BL21-AtIPK	BL21 (DE3) harboring pET-28a-AtIPK	This study
BL21-7-DMATS	BL21 (DE3) harboring pETDuet-7-DMATS	This study
BL21-AtIPK- KJE8	BL21 (DE3) harboring pET-28a-AtIPK and pG-KJE8	This study
BL21-AtIPK-pGro7	BL21 (DE3) harboring pET-28a-AtIPK and pGro7	This study
BL21-AtIPK-pKJE7	BL21 (DE3) harboring pET-28a-AtIPK and pKJE7	This study
BL21-AtIPK-pTf16	BL21 (DE3) harboring pET-28a-AtIPK and pTf16	This study

^a^ TaKaRa Bio, Beijing, China. ^b^ New England Biolab, Beijing, China.

## Data Availability

Not applicable.

## Supplementary Materials

The following supporting information can be downloaded at: https://www.mdpi.com/article/10.3390/ijms232112904/s1.
