# Supplementary material for: Functional Characterization and Screening of Promiscuous Kinases and Isopentenyl Phosphate Kinases for the Synthesis of DMAPP via a One-Pot Enzymatic Cascade
Source: Int J Mol Sci. 2022 Oct 26;23(21):12904. doi: 10.3390/ijms232112904 (PMC9654404; doi:10.3390/ijms232112904)
Supplement: Supplementary file 1 [file ijms-23-12904-s001.zip › ijms-1909472-supplementary.pdf]

SUPPORTING INFORMATION

Functional Characterization and Screening of Promiscuous Kinases and Isopentenyl Phosphate Kinases for the Synthesis of DMAPP via a One-Pot Enzymatic Cascade

Cong Qiu [1,2,†], Yang Liu [1,2,†], Yangbao Wu [1,2], Linguo Zhao [1,2,*] and Jianjun Pei [1,2,*]

[1] Jiangsu Co-Innovation Center of Efficient Processing and Utilization of Forest Resources, College of Chemical Engineering, Nanjing Forestry University, Nanjing 210037, China
[2] Jiangsu Key Lab of Biomass-Based Green Fuels and Chemicals, Nanjing 210037, China
* Correspondence: njfu2302@163.com (L.Z.); peijianjun@njfu.edu.cn (J.P.); Tel.: +86-025-85427962 (L.Z. & J.P.)
† These authors contributed equally to this work.

Table S1. *Km* and *Kcat* values of SfPK, EcPK, ScPK, MtIPK, and MthIPK

Enzyme	*Km* (mM)	*Kcat* (s^{-1})	*Kcat*/*Km* (s^{-1} M^{-1})
SfPK	0.016	0.11	6875
EcPK	0.93	0.063	67.7
ScPK	1.25	0.98	784
MtIPK	0.067	0.027	402.9
MthIPK	0.116	0.0117	100.9

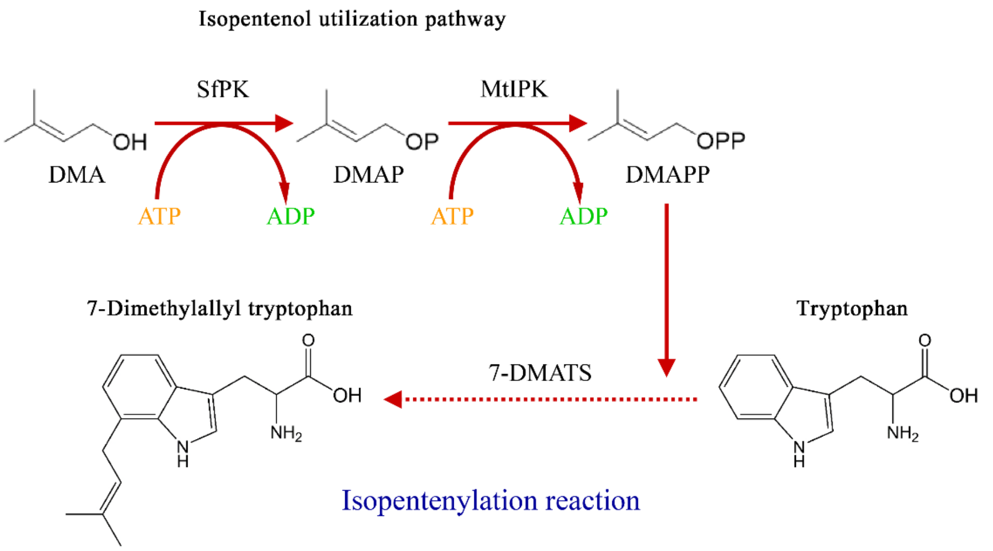

Figure S1. The diagram of DMAPP production by isopentenol utilization pathway in vitro and its detection by prenylating tryptophan.

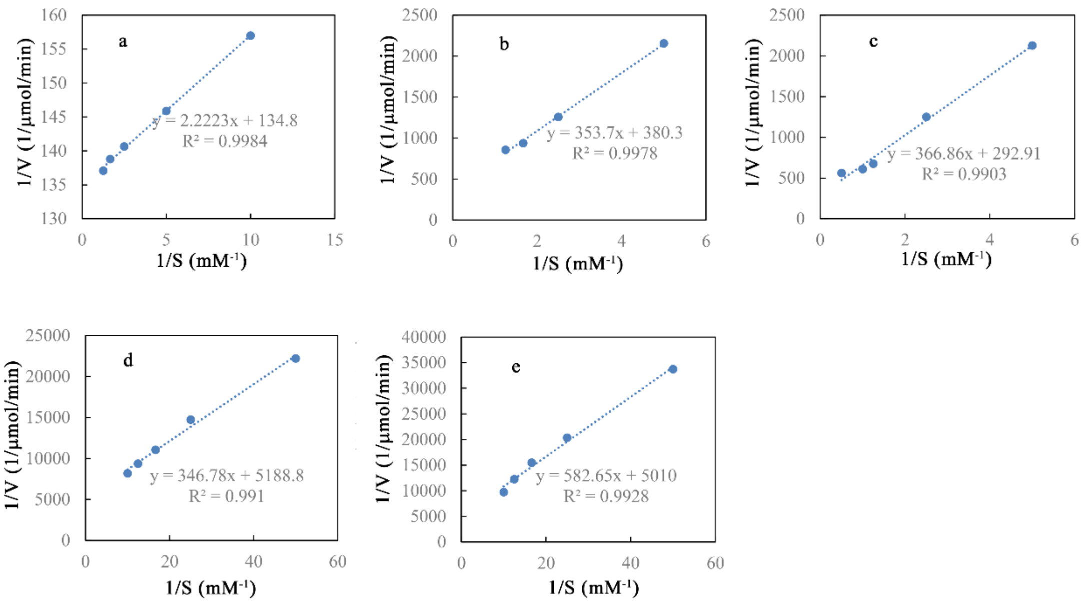

Figure S2. Double-reciprocal plots for SfPK (a), EcPK (b), ScPK (c), MtIPK (d), and MthIPK (e).

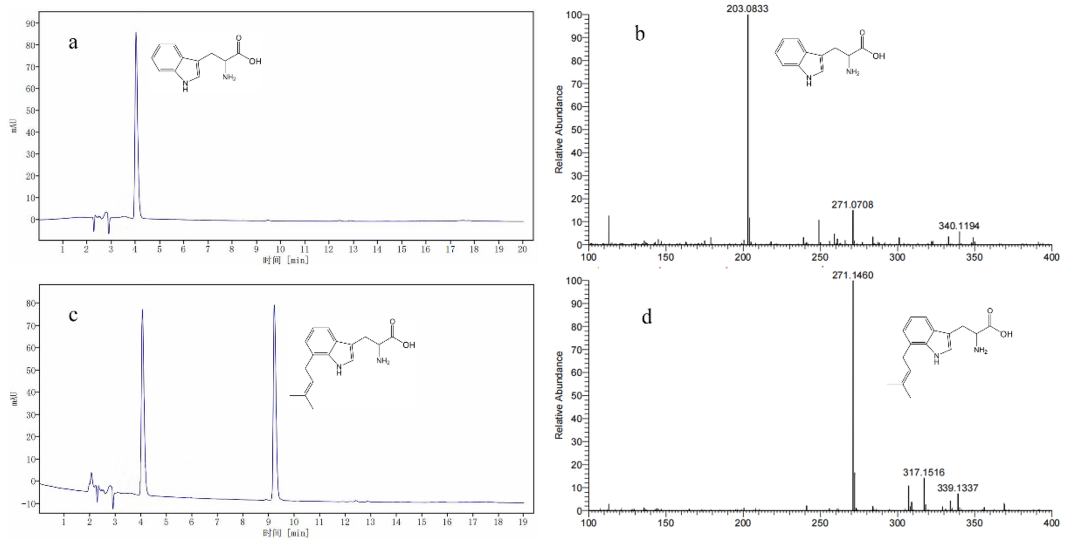

Figure S3. High-performance liquid chromatography (HPLC) and liquid chromatography-mass spectrometry (LC/MS) analyses of 7-dimethylallyl tryptophan and tryptophan. (a) Authentic tryptophan. (b) LC/MS analysis of tryptophan. (c) HPLC analysis of the transformation in vitro by prenyltransferase 7-DMATS, sfPK, and MtIPK. (d) LC/MS analysis of the transformation in vitro by prenyltransferase 7-DMATS, sfPK, and MtIPK.
